# Localized attacks on spatially embedded networks with dependencies

**DOI:** 10.1038/srep08934

**Published:** 2015-03-11

**Authors:** Yehiel Berezin, Amir Bashan, Michael M. Danziger, Daqing Li, Shlomo Havlin

**Affiliations:** 1 Department of Physics, Bar Ilan University, Ramat Gan 52900, Israel; 2 Channing Division of Network Medicine, Brigham and Women's Hospital and Harvard Medical School, Boston, MA, USA; 3 School of Reliability and Systems Engineering, Beihang University - Beijing 100191, China; 4 Science and Technology on Reliability and Environmental Engineering Laboratory - Beijing 100191, China

## Abstract

Many real world complex systems such as critical infrastructure networks are embedded in space and their components may depend on one another to function. They are also susceptible to geographically localized damage caused by malicious attacks or natural disasters. Here, we study a general model of spatially embedded networks with dependencies under localized attacks. We develop a theoretical and numerical approach to describe and predict the effects of localized attacks on spatially embedded systems with dependencies. Surprisingly, we find that a *localized* attack can cause substantially more damage than an equivalent *random* attack. Furthermore, we find that for a broad range of parameters, systems which appear stable are in fact metastable. Though robust to random failures—even of *finite* fraction—if subjected to a localized attack larger than a critical size which is independent of the system size (i.e., a *zero* fraction), a cascading failure emerges which leads to complete system collapse. Our results demonstrate the potential high risk of localized attacks on spatially embedded network systems with dependencies and may be useful for designing more resilient systems.

Many modern critical infrastructures are embedded in two dimensional space^1^,^2^,^3^,^4^,^5^. Examples include ground transportation systems like road and railway networks, electrical power networks, gas and oil pipelines, water supply, the internet and communication lines. The main feature of these spatial networks is that their links represent real physical connections (connectivity links), where link length is relatively short compared to the system size. With the ongoing technological development, these systems have become more and more integrated and interdependent (via dependency links) upon each other. These dependencies lead to substantially increased vulnerability of spatial as well as non-spatial networks to random failures and even first order transitions which are characterized by the emergence of cascading failures^6^,^7^,^8^,^9^,^10^,^11^,^12^,^13^,^14^,^15^,^16^,^17^,^18^,^19^,^20^,^21^. However, failures in spatially embedded systems are often not random but geographically localized. These “localized attacks” can be caused by natural disasters (e.g., the 2011 Tōhoku earthquake and tsunami) or malicious attacks (e.g., weapons of mass destruction). The resilience of a complex system with dependencies under attack of this sort, which we call “localized attack,” has not been addressed before.

Even though different infrastructure systems have their own specific function and dynamics, they share a 2D spatial embedding that implies a fundamental restriction on their structure due the length limitation of connectivity and dependency links. Therefore we study here the general vulnerability of spatially embedded systems under localized attacks. We find here that localized attacks on spatially embedded systems with dependencies are significantly more damaging than random failures (see Fig. 1a), in marked contrast to single networks.

Furthermore, we discover a metastable phase which spans a broad range of parameters and is qualitatively different from the stable and unstable phases known to percolation theory. In metastable systems, there exists a critical damage size with radius 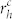, above which localized damage will spread and destroy the entire system and below which the damage will remain in place (see Fig. 1a and 1c). This critical size is determined solely by intensive system quantities and thus, in marked contrast to random failures, it does not scale with system size and constitutes a *zero-fraction* of the system in the limit of large systems (*N* → ∞) (Fig. 1d).

To our knowledge, this is the first study to consider geographically localized attacks from the perspective of percolation theory. Previous research using percolation theory has studied the effects of attacks targeting nodes with special topological properties such as degree but not geographically correlated attacks^22^,^23^,^24^,^25^. Geographic localized attacks have been utilized to identify the most vulnerable parts of specific infrastructure networks^26^,^27^,^28^ but have not been studied in a general percolation framework. Cascading failures have been studied as the outcome of specific dynamic models: load-shedding^29^, binary decisions with fractional thresholds^30^, and betweenness-based loads^31^,^32^. Recently, it was shown that a new kind of cascading failure emerges from percolation on interdependent networks^12^,^13^,^14^,^15^,^16^,^17^,^18^,^19^,^33^. However, these studies considered random attacks only. The unique cascading failures that we describe here have not been observed before. Indeed, they can only arise when the more realistic features of spatial embedding, dependencies and localized attacks are considered together.

Though many of the models for complex systems with dependencies assume dependencies between networks, it has been shown that similar effects are present in a single network composed of connectivity and dependency links^34^,^35^,^36^,^37^. Here, we treat dependency as a general property and examine cascading failures triggered by localized attacks within a single network as well as between networks.

When considering spatially embedded networks, the dimension of a network is a fundamental quantity to characterize its structure and basic physical properties^39^. On the basis of universality principles, all single network models with links of a characteristic length, embedded in a space of the same dimension, have the same percolation behavior^38^. Therefore, any 2D network with a characteristic link length belongs to the same universality class as regular lattices. When dependency links are introduced, the critical behavior is additionally determined by the length of the dependency link^17^. For tractability, the theory presented in this work is based on 2D lattices. However, the effects of localized attacks on systems with dependencies are expected to be the same for any system embedded in 2D space as illustrated with the European power grid^40^ in Sup. Fig. 2 and synthetic power grids^41^,^42^ in Supplementary Figs. 1–4.

We model spatially embedded systems via square lattices diluted to degree 2.5 ≤ 〈*k*〉 ≤ 4. The dependencies between nodes are constrained to be less than a distance *r* (in lattice units) and can be taken across networks or within a single network. See *Methods* for details of system construction.

The localized geographical attack is modeled by the removal of all nodes within a distance *r_h_* from a random location in the system (see Fig. 1a–b). This triggers a cascade in which the nodes that depend on the removed nodes fail, triggering further losses as more nodes get cut off from the largest connected component. This percolative damage triggers further damage due to the dependencies between the nodes. This process is continued iteratively until no more nodes fail. At the end of this cascade, the system is categorized as functional or non-functional depending on whether a largest connected component of the order of the system size *N* remains or not.

## Results

**Figure 1:**
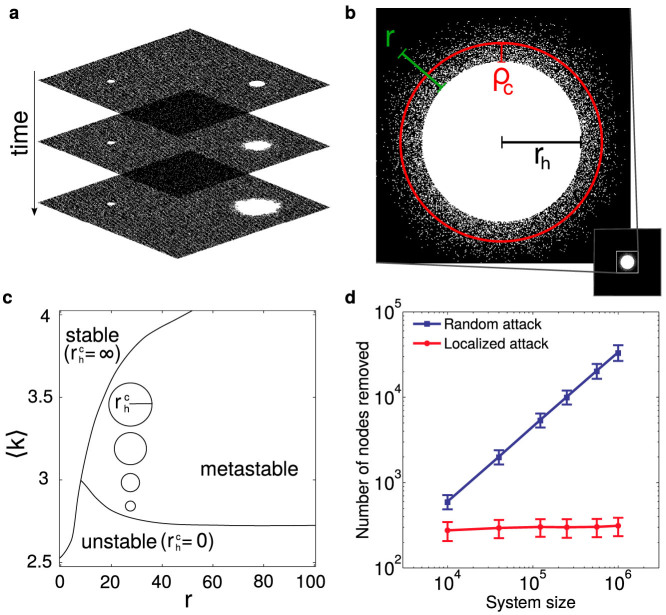
The effect of a localized attack on a system with dependencies. (a), Propagation of local damage in a system of two interdependent diluted lattices with spatially constrained dependency links between the lattices (only one lattice shown here). The hole on the right is above the critical size 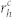 and spreads throughout the system while the hole on the left is below 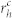 and remains essentially the same size. (b), A localized circular failure of radius *r_h_* in a lattice with dependency links of length up to *r*. Outside the hole, the survival probability of a node increases with the distance *ρ* from the edge. The parameter *ρ_c_* denotes the distance from the edge of the hole at which the occupation probability is equal to the percolation threshold of a lattice without dependencies *p_c_* ≈ 0.5927^36^. (c), Phase diagram of a lattice with dependencies or two interdependent lattices. Depending on the average degree 〈*k*〉 and dependency length *r*, the system is either stable, unstable or metastable. The circles illustrate the increase (when 〈*k*〉 increases) of the critical attack size (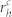) that leads to system collapse in the metastable region. (d), As the system size grows, the minimal number of nodes which cause the system to collapse increases linearly for random attacks but stays constant (≈300) for localized attacks. This figure was obtained for a system of interdependent lattices diluted to 〈*k*〉 ≈ 2.9 and *r* = 15 (in the metastable phase-see c), with 1000 runs for each data point.

We discover that the *k*–*r* plane can be divided into three distinct phases as shown in Fig. 1c. In the stable phase, no matter how large *r_h_* is (as long as it is finite) the damage will remain localized and the system will stay intact. In the unstable phase, the system spontaneously collapses even with *r_h_* = 0 (no localized attack). In this phase, low 〈*k*〉 and dependencies lead to the spontaneous emergence of holes which overwhelm the system. Between these phases, the system is metastable. If a hole smaller than 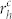 is removed, the system remains intact. However, if a hole of size 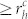 is removed, it will trigger a cascade which destroys the entire system. This cascade is characterized by the spread of damage from the initial localized attack throughout the system (Fig. 1a, b). This metastability is analogous to the well known supercooling property of water in which water can be cooled well below its freezing point and remain in the liquid state until a disturbance triggers crystallization of a critical size and it turns to ice^44^.

For a system in the metastable phase under *random* attack, the number of nodes required to trigger system collapse increases linearly with the system size (See Fig. 1d). Therefore, as *N* → ∞, metastable systems are robust to the removal of *O*(*N*) nodes, as long as they are removed randomly. However, if the attack is *localized*, the number of nodes required remains constant (Fig. 1d) and even a zero fraction removed can trigger a cascading failure which destroys the system. Thus increasing the size of the system does not increase its resilience with respect to localized attacks. We find similar results for both interdependent networks and single networks composed of connectivity and dependency links. The results presented in the main text were obtained from interdependent networks and comparison to single networks with connectivity and dependency links is shown in Supplementary Fig. 1.

Predicting the value of 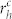 for a given system is an important question which is treated below theoretically and numerically, with good agreement.

### Simulations

**Figure 2:**
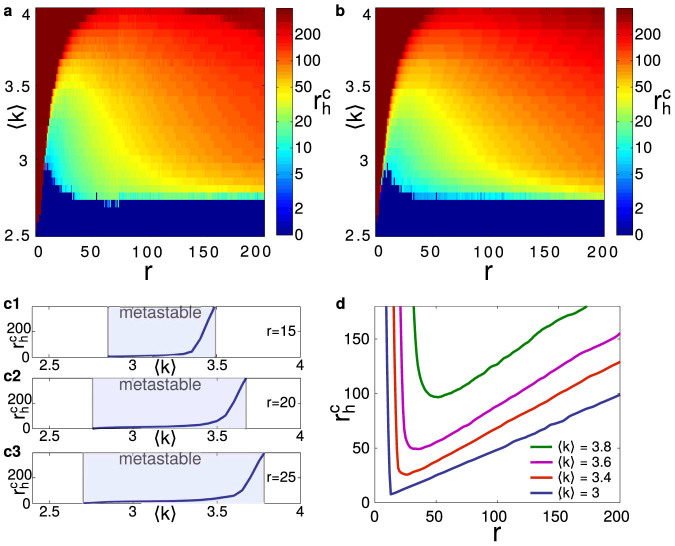
Dependence of the critical attack size 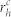 on the average degree 〈*k*〉 and the system dependency length *r*. (a, b), The value of 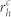 as a function of the dependency length *r* and average degree 〈*k*〉 represented as a log-scaled colormap. (a), Simulation results. We use a binary search algorithm to find the critical radius size, ie, the minimal *r_h_* for which the local attack spreads through the entire system. (b), Analytical results. The critical size is calculated as the smallest value of *r_h_* for which Eq. (3) has a self-consistent solution. For a numerical comparison between the simulation and analytical results in the metastable phase, see Supplementary Fig. 5. (c1, c2, c3), Critical attack size 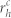 as a function of average degree 〈*k*〉 for three *r* values, determined by simulations. The curves represent moving along vertical lines from bottom to top in (a) (cf. the circles in Fig. 1c). The shaded region represents the metastable region of 〈*k*〉 for each *r*. The area to the left of the shaded region is unstable and to the right is stable. (d), Critical attack size 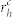 as a function of system dependency length *r* for several 〈*k*〉 values, determined by simulations. The minimum of each curve represents the dependency length for which the system is most vulnerable to localized attacks. The numerical results in this figure were generated using a system of two interdependent diluted lattices. For comparison with a single diluted lattice composed of both, connectivity and dependency links, see Supplementary Information Fig. 1.

We find that 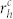 is entirely determined by the average degree 〈*k*〉 and the maximal dependency link length *r*. These are intensive system quantities and therefore 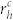 does not grow with system size (Fig. 1d). Figs. 2c and 2d show how the critical damage size 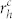 changes with respect to 〈*k*〉 and *r* for a system of size *L* × *L* = 1000 × 1000. In Fig. 2c we can see that the metastable region covers a wider range of 〈*k*〉 values when *r* increases. In the metastable phase, for every *r*, 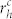 increases with 〈*k*〉 and jumps up dramatically at a certain 〈*k*〉 value which represents the end of the metastable phase and the beginning of the stable phase. Furthermore, we see that this jump occurs at larger 〈*k*〉 values for larger *r* values (Fig. 2c). In Fig. 2d, we see that above a certain minimum value, 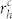 has an approximately linear dependence on *r* in the metastable region. This is due to the fact that a larger *r* means that a given node's dependency link can be located farther away. Thus the secondary damage from the localized attack is more dispersed and a larger attack size is required to initiate a cascade. Furthermore, we find that the critical damage size 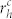 takes a minimal value and the system is most susceptible to small local attacks when *r* is near the stable phase. Extensive numerical simulations of 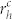 over a high resolution grid of parameters 〈*k*〉 and *r* is shown in Fig. 2a and the theoretical prediction which is in good agreement is given in Fig. 2b. The theoretical description of the effect of 〈*k*〉 and *r* on 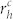 is presented below.

### Theory

Since the metastable region spreads over a wide range of realistic values of *r* and 〈*k*〉, it is of great interest to understand how this transition takes place and to develop a theory to predict the value of 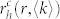. To do so, we first consider in detail the chain of events triggered by the geographically localized damage. When a hole of *r_h_* is removed from the system, it can directly disable nodes up to a distance *r* from its edge due to the existence of dependency links of length ≤ *r* (see Fig. 1b). The probability that a given node was dependent on one of the removed nodes is highest at the edge of the hole and monotonously decreases with the distance from the edge, until it equals zero at distance *r*. To calculate this decrease, we need to calculate the probability that a node *i* depends on a node which was removed in the localized attack (cf. Fig. 1b). This probability is determined by the area of intersection of two circles: the localized attack (with radius *r_h_*) and the circle of maximal dependence (with radius *r* and center *i*). Taking *ρ* as the distance from the edge of the hole, the gradient of occupation probability following an attack can be evaluated as
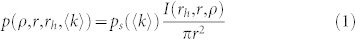
where *p_s_*(〈*k*〉) is the occupation concentration before the attack and *I*(*r_h_*, *r*, *ρ*) is the standard formula for the area of intersection of two circles of radius *r* and *r_h_* with centers located a distance *ρ* + *r_h_* from each other. This probability describes a lattice concentration gradient in the form of an annulus of width *r* surrounding the removed hole, see Fig. 1b. For a given set of system parameters (*r*, *r_h_*, 〈*k*〉) we can set *p*(*ρ*) on the LHS of Eq. (1) to *p_c_* of the lattice and solve for *ρ*. If a solution in the region of interest (0 < *ρ* < *r*) exists, it corresponds to a distance *ρ_c_* at which the lattice concentration will be equal to its critical value. The existence of such a point is a necessary but not sufficient condition for the hole to propagate. Below *p_c_*, the network forms clusters with a characteristic size *ξ*_<_(*p*), which diverges at *p_c_*, where *ξ*_<_(*p*) is the connectedness correlation length for *p* < *p_c_*^38^,^45^. Hence the sufficient condition for damage propagation is that the critical region 0 < *ρ* < *ρ_c_* be wide enough for clusters of size *ξ*_<_(*p*) to form and break away.

The value of *ξ*_<_(*p*) is determined by the underlying topology and can thus be calculated from the percolation problem on a lattice without dependencies using an appropriate estimation for *p* in the 0 < *ρ* < *ρ_c_* region. From Eq. (1), *p*(*ρ*) increases monotonically over this region and an accurate evaluation solution for *ξ*_<_ would require treating the full gradient percolation problem^46^. In this work, for simplicity we assume 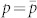 which is the average of *p*(*ρ*) over the region of interest. Additionally, the removal of the hole causes secondary damage due to dependencies in the annulus and the concentration of the gradient is decreased by a factor of *g*(*r*) which we calculate numerically and find to vary monotonically from 0.85 to 0.89 as a function of *r*. We can thereby estimate 
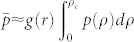
. We evaluate *ξ*_<_ following^38^ as:
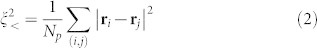
where (*i*, *j*) refers to nodes *i* and *j* which are in the same connected component, *r_i_* is the coordinate and, *N_p_* is the total number of such pairs of nodes. In order for the hole to propagate, the clusters which are of typical size *ξ*_<_, need to be smaller than *ρ_c_*. Therefore, the critical hole size (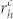) for any system is obtained from the self-consistent solution of
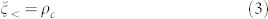
where both sides are functions of *r*, *r_h_* and *p_s_*. Using these considerations, we can predict 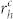 for every set of (*k*, *r*) parameters as shown in Fig. 2b. These theoretical results are in close agreement with the numerical simulations (Fig. 2a) for the value of 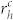 as well as its lack of scaling with system size (Fig. 1d). Though the above method for calculating 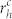 is accurate only for systems of diluted lattices, the effects of local attacks on more realistic topologies are the same as shown on the UCTE European power grid^40^ and on synthetic power grids^41^ in Supplementary Figs. 2–4.

## Discussion

Everything about the scenario described above is local: nodes can have dependency links only up to length *r*, the connectivity links are tied to an underlying lattice structure with characteristic length of one unit in the model or are limited by length-cost in the real world system and the attack is restricted to a hole of finite radius *r_h_*. However, for a wide range of system parameters, this leads to a catastrophic cascade which destroys the entire system.

It is instructive to compare this process to a single spatially embedded network without dependencies. If a hole of any finite size is created in a lattice or other spatially embedded network, it will have no effect on the overall system robustness. Only the trivial case of *r_h_* approaching the system size *L* leads to system collapse. A similar argument holds with respect to dependency links which are not restricted in length. If a hole of size *r_h_* (mass ~ 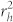) is removed from one network, it will lead to *random* removal of a fraction ~ 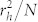 in the other network. Therefore, in the limit of large systems, the numerator remains constant while the denominator tends to infinity and we find that here too the localized attack will have negligible impact. Only when the dependency links are of limited length does this unique phenomenon arise.

Surprisingly, the localization of dependency opens the door for the spreading phenomenon which amplifies the local damage and leads to total system collapse. When a hole of radius *r_h_* is removed from the system, the nodes that depended on them must be within a distance *r* of the hole. Thus the secondary damage is highly concentrated around the edge of the hole, leading to the creation of a damage front which propagates outwards, step by step. This is why the amount of damage caused per node removed is substantially higher when the damage is localized as compared with random removal (Fig. 1d). If *r* → ∞ or *r* → 0, this weakness would not exist because the secondary damage would spread everywhere uniformly or remain in place, respectively.

Paradoxically, the highly localized topology of embedded interdependent networks enables relatively small localized attacks to cause catastrophic global damage. These results have profound implications for the role of network topology in the design of resilient infrastructures.

We note that after the submission of this manuscript, an analytical framework to study localized attacks on non-embedded networks was developed by Shao et al.^43^

## Methods

On the basis of universality principles, the theoretical analysis and specific predictions for 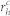 presented in this work are based on a lattice model. To make the model more realistic while maintaining its solvability, we have diluted the lattices from the standard square lattice (*k* = 4) to lower values of 〈*k*〉. The range of 〈*k*〉 values studied here is based on empirical studies of power grids which have found a mean degree of 2.5 ≤ 〈*k*〉 ≤ 3^42^. This dilution is carried out by removing a given fraction of nodes from the system and allowing the percolative process to reach a steady state, including the effects of the dependencies. This dilution process is equivalent to the percolation problem on interdependent spatially embedded networks^17^.

Dependencies can be constructed between networks or within a network. We model dependencies between spatially embedded networks by overlaying two diluted square lattices *A* and *B* of size *L* × *L* with periodic boundary conditions on the same Cartesian plane. Each node in network *A* is dependent upon a node in network *B* (and vice versa) which is chosen at random from all of the nodes within a radius *r*. If a node in *A* is dependent on a node in *B*, the failure of the node in *B* will cause the node in *A* to fail immediately and vice versa. These dependency relationships are taken to be mutual to prevent a single failure from propagating through the entire system^16^. The numerical results in this paper were generated in this manner. The lattice size was *L* = 1000 (*N* = 10^6^), 〈*k*〉 was sampled in intervals of 0.05 and *r* sampled in intervals of one lattice unit.

We obtain the same results via constructing dependencies between nodes on a single network in the following way. For each node *i*, a random node *j* within a radius *r* from *i* is selected and the two nodes are taken to be mutually interdependent. The exposition of the theory above describes the effects of dependencies of either type. The numerical results are essentially the same, see Supplementary Fig. 1 for a detailed comparison.

## Author Contributions

Y.B., A.B., M.M.D., D.L. and S.H. conceived and designed the research. Y.B. carried out the numerical simulations. Y.B., A.B. and M.M.D. developed the theory and wrote the paper with contributions from all other authors. The authors declare no competing financial interests.

## Supplementary Material

Supplementary Information Supplementary Information
