# Supplementary material for: Localized attacks on spatially embedded networks with dependencies
Source: Sci Rep. 2015 Mar 11;5:8934. doi: 10.1038/srep08934 (PMC4355725; doi:10.1038/srep08934)
Supplement: Supplementary Information [file srep08934-s1.pdf]

Localized attacks on spatially embedded networks with dependencies: Supplementary Information

Yehiel Berezin,[1] Amir Bashan,[2] Michael M. Danziger,[1] Daqing Li,[3,4] and Shlomo Havlin[1]

[1]*Department of Physics, Bar Ilan University, Ramat Gan 52900, Israel*

[2]*Channing Division of Network Medicine, Brigham and Women's Hospital and Harvard Medical School, Boston, MA, USA.*

[3]*School of Reliability and Systems Engineering, Beihang University - Beijing 100191, China*

[4]*Science and Technology on Reliability and Environmental Engineering Laboratory - Beijing 100191, China*

(Dated: December 23, 2014)

I. LOCALIZED ATTACKS ON A PAIR OF COUPLED NETWORKS COMPARED TO A SINGLE NETWORK WITH DEPENDENCY AND CONNECTIVITY LINKS

The numerical results in Fig. 3 of the manuscript were generated on a system of a pair of interdependent lattices. However, the theory presented in the manuscript also applies to systems of single networks having both connectivity and dependency links [4–6]. To demonstrate that this is the case we compared the values of r_h^c directly for a range of $\langle k \rangle$ and r values in single and coupled networks. As is evident in Sup. Fig. 1, the values are almost identical. Small differences exist in the values due to the fact that in simulating the localized attack on the interdependent networks, a hole was removed from only one of the networks. Under such an attack in a system of two networks, the nodes in the second network at the same position as the attacked nodes have a finite probability of survival, increasing with $\langle k \rangle$ and $(r - r_h^c)$.

II. LOCALIZED ATTACKS ON REAL-WORLD POWER GRIDS

The theory and simulations presented in this manuscript describes the effects of local attacks on any network with a characteristic length scale. The specific calculations for the critical attack size, r_h^c which we presented are based on a system of diluted lattices. To demonstrate the relevance of spatially localized attacks on more realistic topologies, we simulated local attacks on the UCTE EU power grid [7] (See Sup. Fig. 2) and on synthetic power grids (see Sup. Fig. 3).

Though the topologies of these systems differ markedly from diluted lattices (see Sup. Figs. 3 and 4), they too are metastable and localized damage of size $> r_h^c$ triggers a spreading cascade which destroys the entire system. Because the distance distributions for power grids are exponential (Sup. Fig. 4), they have a characteristic length scale which puts them in the same universality class as lattices [8]. Furthermore, the vast majority of links are very short and thus the damage from a localized attack is

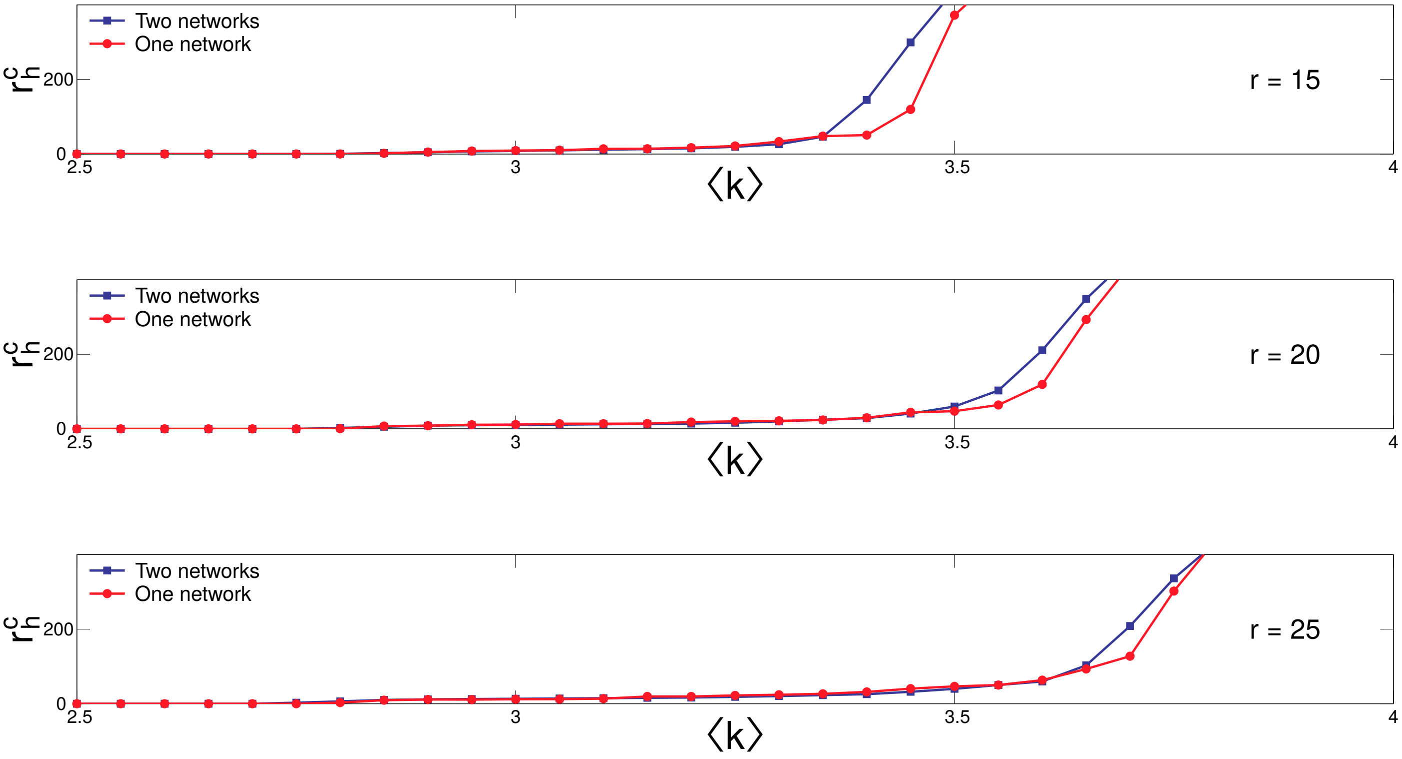

Supplementary Figure 1: **Comparison between single networks and coupled networks: critical hole size r_h^c as a function of $\langle k \rangle$.** The values of r_h^c are the same for two networks and one network apart from small deviations for large $\langle k \rangle$ values. The reason for this deviation is that the hole is removed from only one network in the two network system and there is thus a finite probability (increasing with $\langle k \rangle$) that the nodes at the same coordinates in the second network will remain functional. In a single network, this effect does not exist and therefore r_h^c is slightly smaller. This effect decreases when the dependency length r increases.

indeed concentrated along its perimeter and a propagating front can appear in these systems as well.

III. SYNTHETIC POWER GRID GENERATION

The synthetic power grids shown in Supplementary Figs. 3 and 4 were generated following a modified version of the generative growth model published by Deka and Vishwanath [9]. Rather than beginning the generative model on an empty plane, we begin with a portion of the nodes present and then follow Deka and Vishwanath's procedure. This is effectively a hybrid model composed of Deka's generative model [9] with Hines static model [1] and this allows for the reproduction of a power grid that is connected and has physically reasonable distributions for geographic link length and degree (see Sup. Fig. 4).

In Fig. 2 we demonstrate the effects of local attacks on the UCTE European power grid.

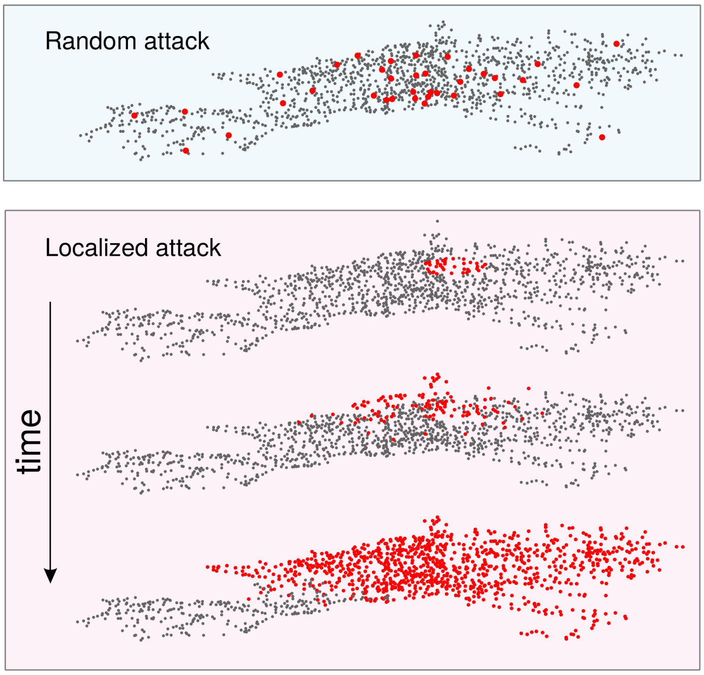

Supplementary Figure 2: **Temporal snapshots of damage propagation in systems with dependencies.** Comparison between a *random* attack and a *localized* attack of the same size (37 nodes removed–less than 3% of the system) on a networks with the same structural layout of the European power grid [7] with spatially constrained dependencies ($r \approx 500km$) between different nodes. The random attack does not cause substantial further damage while the localized attack (of the same number of nodes) triggers a cascade that overwhelms the entire system. (Number of nodes $N = 1254$, $\langle k \rangle = 2.88$).

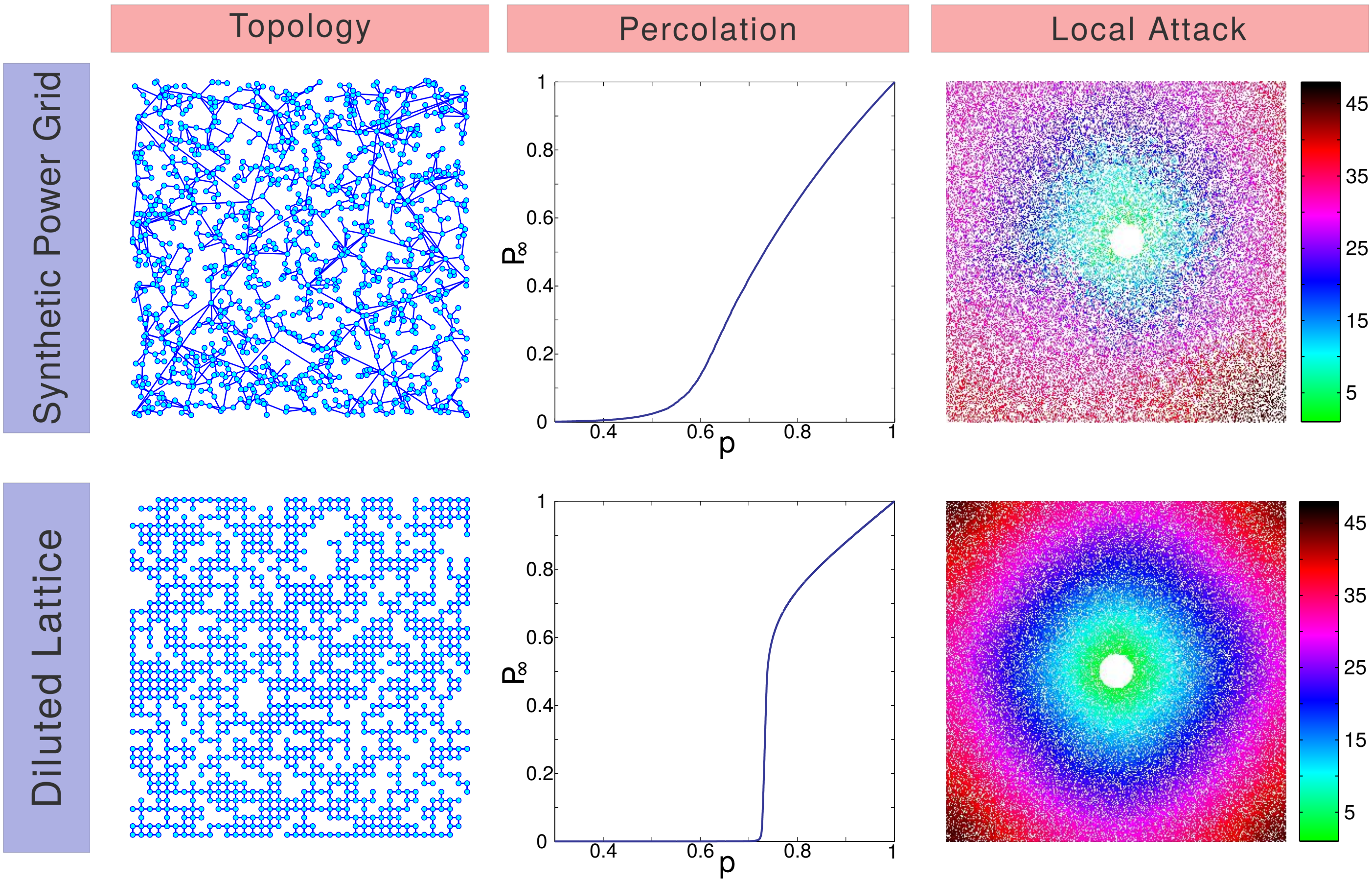

Supplementary Figure 3: **Comparison of diluted lattices and synthetic power grids.** Though diluted lattices have different topology (left column) and percolation behavior (middle column) from synthetic power grids, the response to a local attack is essentially the same (right column). The colors in the local attack section (right column) of the figure represent the time steps at which the nodes failed (right colorbar).

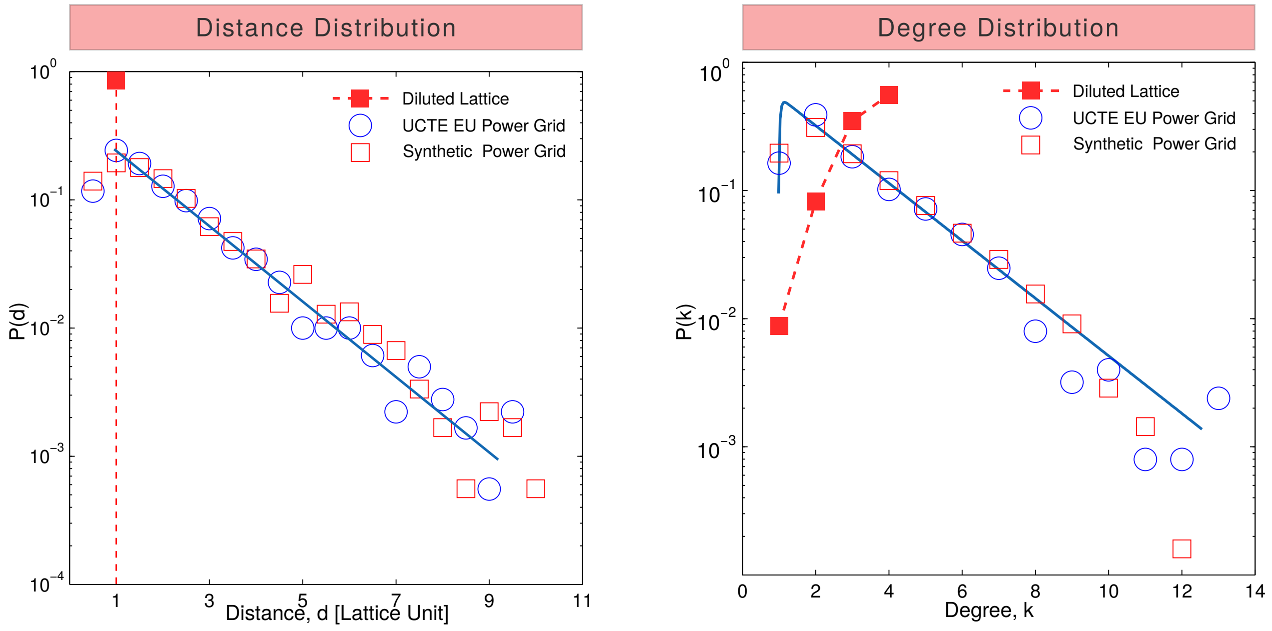

Supplementary Figure 4: **Comparison of degree and link length distributions between diluted lattices and power grids.** The power grids have substantially different degree and geographic distance (link length) distribution properties. The link length and degree distributions for power grids have exponential tails. However, since the majority of the power grid links are low-distance, the spreading phenomenon caused by local attacks still takes place.

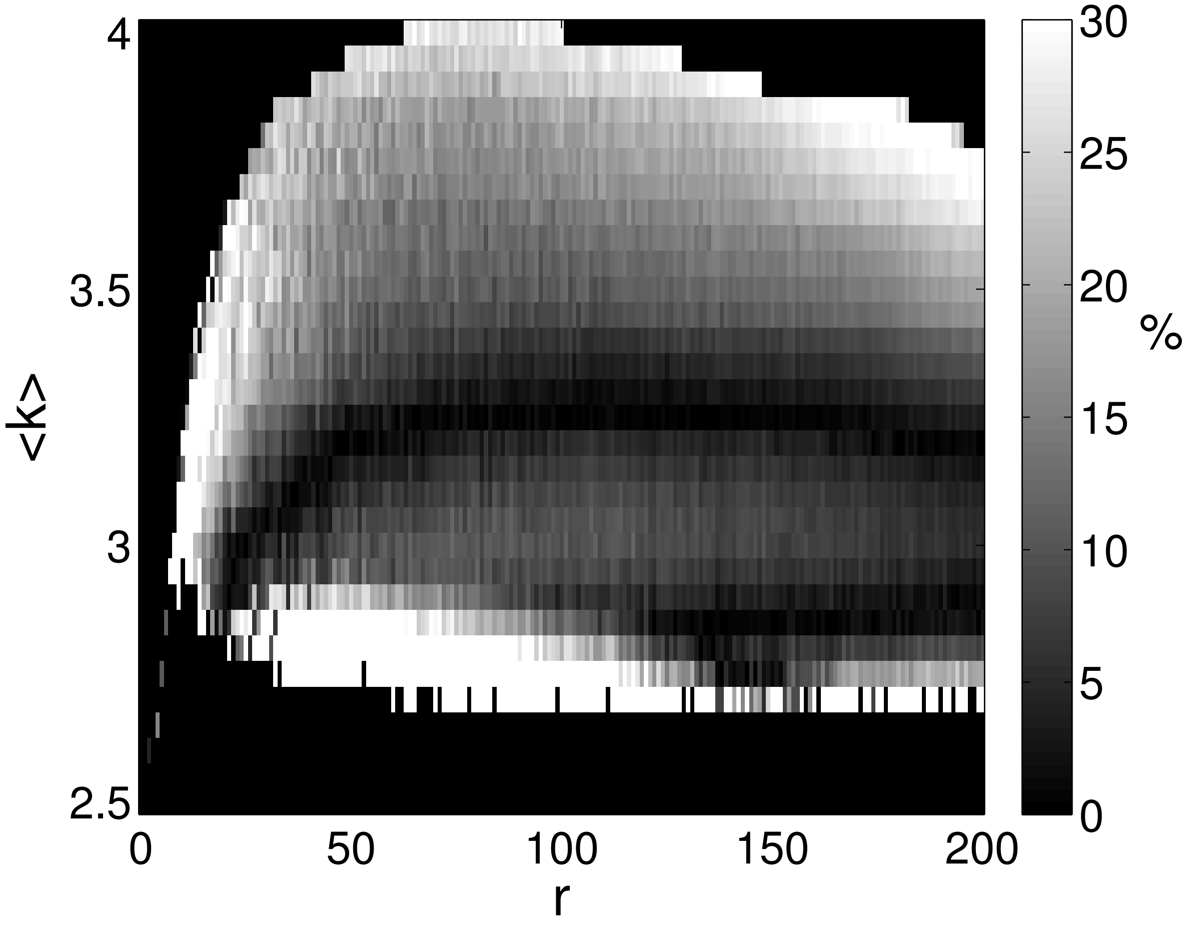

Supplementary Figure 5: **Relative error of r_h^c calculation in the metastable phase.** This figure shows the difference between the calculated and simulated r_h^c values, divided by the calculated value, in the metastable phase. For comparison of the phase boundaries, we refer the reader to Fig. 2a-b in the main text. The agreement between the theory and simulation is very close for the bulk of the metastable phase.

[1] Hines, P. *et al.* The Topological and Electrical Structure of Power Grids. In *System Sciences (HICSS), 2010 43rd Hawaii International Conference on*, 1–10 (2010).

[2] Li, W. *et al.* Cascading Failures in Interdependent Lattice Networks: The Critical Role of the Length of Dependency Links. *Phys. Rev. Lett.* **108**, 228702 (2012).

[3] Gao, J. *et al.* Networks formed from interdependent networks. *Nature Physics* **8**, 40–48 (2012).

[4] Parshani, R., Buldyrev, S. V. & Havlin, S. Critical effect of dependency groups on the function of networks. *Proceedings of the National Academy of Sciences* **108**, 1007–1010 (2011).

[5] Bashan, A., Parshani, R. & Havlin, S. Percolation in networks composed of connectivity and dependency links. *Phys. Rev. E* **83**, 051127 (2011).

[6] Zhao, J.-H., Zhou, H.-J. & Liu, Y.-Y. Inducing effect on the percolation transition in complex networks. *Nature Communications* **4** (2013).

[7] Zhou, Q. & Bialek, J. Approximate model of european interconnected system as a benchmark system to study effects of cross-border trades. *Power Systems, IEEE Transactions on* **20**, 782–788 (2005).

[8] Li, D. *et al.* Dimension of spatially embedded networks. *Nature Physics* **7**, 481–484 (2011).

[9] Deka, D. & Vishwanath, S. Generative growth model for power grids. In *2013 International Conference on Signal-Image Technology & Internet-Based Systems*, 591–598 (2013).
